# Bovine Lactoferrin Suppresses Tumor Angiogenesis through NF-κB Pathway Inhibition by Binding to TRAF6

**DOI:** 10.3390/pharmaceutics15010165

**Published:** 2023-01-03

**Authors:** Nurina Febriyanti Ayuningtyas, Chanbora Chea, Toshinori Ando, Karina Erda Saninggar, Keiji Tanimoto, Toshihiro Inubushi, Nako Maishi, Kyoko Hida, Masanobu Shindoh, Mutsumi Miyauchi, Takashi Takata

**Affiliations:** 1 Department of Oral & Maxillofacial Pathobiology, Graduate School of Biomedical & Health Sciences, Hiroshima University, 1-2-3 Kasumi, Minami-ku, Hiroshima 734-8553, Japan; 2 Department of Oral Medicine, Faculty of Dental Medicine, Universitas Airlangga, Prof. Dr. Moestopo 47, Surabaya 60132, Indonesia; 3 Center of Oral Clinical Examination, Hiroshima University Hospital, 1-2-3 Kasumi, Minami-ku, Hiroshima 734-8553, Japan; 4 Department of Conservative Dentistry, Faculty of Dental Medicine, Universitas Airlangga, Prof. Dr. Moestopo 47, Surabaya 60132, Indonesia; 5 Department of Radiation Medicine, Research Institute for Radiation Biology and Medicine, Hiroshima University, 1-2-3 Kasumi, Minami-ku, Hiroshima 734-8553, Japan; 6 Department of Orthodontics and Dentofacial Orthopedics, Graduate School of Dentistry, Osaka University, 1-8 Yamada-Oka, Suita 565-0871, Japan; 7 Department of Vascular Biology and Molecular Pathology, Hokkaido University Graduate School of Dental Medicine, Kita-13, Nishi-7, Kita-Ku, Sapporo 060-8586, Japan; 8 Hokkaido University, Kita-13, Nishi-7, Kita-Ku, Sapporo 060-8586, Japan; 9 Shunan University, 843-4-2 Gakuenndai Syunan, Yamaguchi 745-8566, Japan

**Keywords:** tumor angiogenesis, lactoferrin, TRAF6, VEGF, HIF-1α, cancer

## Abstract

Tumor angiogenesis is essential for tumor progression. The inhibition of tumor angiogenesis is a promising therapy for tumors. Bovine lactoferrin (bLF) has been reported as an anti-tumor agent. However, bLF effects on tumor angiogenesis are not well demonstrated. This study evaluated the inhibitory effects of bLF on tumor angiogenesis in vivo and in vitro. Herein, tumor endothelial cells (TECs) and normal endothelial cells (NECs) were used. Proliferation, migration, tube formation assays, RT-PCR, flow cytometry, Western blotting, siRNA experiments and immunoprecipitation were conducted to clarify the mechanisms of bLF-induced effects. CD-31 immunoexpression was examined in tumor tissues of oral squamous cell carcinoma mouse models with or without Liposomal bLF (LbLF)-administration. We confirmed that bLF inhibited proliferation/migration/tube formation and increased apoptosis in TECs but not NECs. TNF receptor-associated factor 6 (TRAF6), p-p65, hypoxia inducible factor-α (HIF-1α) and vascular endothelial growth factor (VEGF) were highly expressed in TECs. In TECs, bLF markedly downregulated VEGF-A, VEGF receptor (VEGFR) and HIF-1α via the inhibition of p-p65 through binding with TRAF6. Since NECs slightly expressed p-p65, bLF–TRAF-6 binding could not induce detectable changes. Moreover, orally administrated LbLF decreased CD31-positive microvascular density only in TECs. Hence, bLF specifically suppressed tumor angiogenesis through p-p65 inhibition by binding to TRAF6 and suppressing HIF-1α activation followed by VEGF/VEGFR down-regulation. Collectively, bLF can be an anti-angiogenic agent for tumors.

## 1. Introduction

Angiogenesis, a complex multistep process including proliferation, migration, the differentiation of endothelial cells, degradation of the extracellular matrix, microtubule formation, and the sprouting of new capillary branches, is essential for reproduction, development, and repair [1,2,3]. Solid tumors depend on pathological angiogenesis to grow beyond microscopic size (>1–2 mm) and cause bleeding and vascular leakage [4]. The microvascular endothelial cell recruited by a tumor is an important second target in cancer therapy and is genetically stable [5]. Diverse genetic instabilities of cancer cells are a major evolutionary factor for the selection of drug-resistant mutants. The lack of such genetic aberrations in activated endothelial cells results in little drug-resistance development [6]. Therefore, treating both the cancer cell and the endothelial cell in a tumor may be more effective than treating cancer cells alone. Thus, the attempt to target tumor endothelial cells in clinical fields with angiogenic inhibitors has been an important strategy [5].

Vascular endothelial growth factor A (VEGFA) seems important for endothelial cell development [7]. VEGFA exerts multiple effects on angiogenesis [8,9], such as the induction of new blood vessels formation and up-regulation of vascular permeability [10]. VEGFA stimulates endothelial cells, mainly via its receptor FLT1 or FLK1 [11,12]. FLK1 is the most important receptor in VEGFA-induced mitogenesis and permeability, whereas FLT1 has regulatory effects of FLK1 [13,14]. Moreover, VEGFA is one of the downstream targets of hypoxia-inducible factor-1 alpha (HIF-1α), a transcription factor regulating cell response to hypoxia and acting as a regulator of oxygen homeostasis [15]. In the microenvironment of many tumor entities, hypoxia is caused by structural and functional abnormality of vessels and increased oxygen consumption caused by the rapid proliferation of tumor cells [16]. HIF-1α and VEGFA are major regulators of tumor angiogenesis and of tumor progression in many types of cancer [17,18]. Importantly, medications targeting VEGFA have been developed. Although they increase survival in some patients, they also increase the risk of several adverse effects. Since angiogenesis is also a critical phenomenon in physiological condition, the ubiquitous inhibition of angiogenesis may lead to adverse side effects. Therefore, it is required to establish a new anti-angiogenic therapy, which specifically inhibits tumor angiogenesis.

Lactoferrin (LF), an 80-kD iron-binding glycoprotein of the transferrin family with a wide spectrum of biological effects, including anti-bacterial, anti-inflammatory, immunomodulatory and anti-cancer activities, is present in various secretory fluids. Bovine LF (bLF), a protein found in cow’s milk, did not show toxic effects and is safe in doses lesser than 2000 mg/kg body weight/day in adults, according to the European Food Safety Authority (EFSA) Panel [19]. Recently, the LF anticancer effect has been demonstrated in several cancer cells and/or animal models bearing different types of tumors, including lung, colon, melanoma and oral cancer [20,21,22,23,24]. Moreover, bLF has been clinically used in patients with various cancers, including colon cancer, with successful inhibitory effects in clinical trial [25,26,27]. Thus, bLF can be considered an anti-neoplastic agent for human cancers. However, the action mechanisms of bLF in tumor angiogenesis have not been described. The purpose of this study was to clarify the specific inhibitory effects and action mechanisms of bLF on tumor angiogenesis in vitro and in vivo.

## 2. Materials and Methods

### 2.1. Cells and Cell Culture

Tumor endothelial cells (TECs) and normal endothelial cells (NECs) were isolated as previously reported [28,29].

Briefly, TECs were isolated from melanoma (A375SM) xenografts in nude mice (8–12 weeks old, Charles River, Boston, MA, USA). Human A375SM cells (10^6^ cells/mouse), obtained from Dr. Isaiah J Fidler (M.D. Anderson Cancer Center Houston, TX, USA), were injected into the dorsal lateral flanks of female nude mice subcutaneously. When tumors reached 1 cm in diameter, they were excised from 10 nude mice. To obtain NECs, the dermal tissue on the back of 10 female mice of non-tumor bearing were excised.

Excised tissues were minced and digested with collagenase II (Worthington, Freehold, NJ, USA). Blood cells were removed by a single sucrose step-gradient centrifugation with Histopaque 1077 (Sigma-Aldrich), and cell suspensions were filtered. Endothelial cells were isolated using MACS (Miltenyi Biotec, Auburn, CA, USA) [28], according to the manufacturer’s instructions using FITC-anti-CD31 antibody. CD31-positive cells were sorted and plated onto 1.5% gelatin-coated culture plates and grown in EGM-2MV (Clonetics, Walkersville, MD, USA) and 10% fetal calf serum. Diphtheria toxin (500 ng/mL; Calbiochem, San Diego, CA, USA) was added to endothelial cell subcultures to eliminate any remaining human tumor cells that expressed heparin-binding EGF-like growth factor, a DT receptor. After 24 h, dead cells were aspirated. Endothelial cells were purified by a second MACS using FITC-BS1-B4 to eliminate contaminating stromal cells at 2 weeks of subculture and were cultured in EGM-2MV. The purity of endothelial cells was confirmed by flow cytometric analysis of cell surface protein with FITCBS1- B4. TECs and NECs were cultured in a collagen type I-coated dish (Celltight C-1, Sumitomo Bakelite Co., Tokyo, Japan) containing EGM^TM^-2 MV (Lonza, Basel, Switzerland) with 10% fetal bovine serum (FBS) HyClone (Thermo Fisher Scientific, Pittsburgh, PA, USA) under normoxic conditions (5% CO_2_ and 95% air at 37 °C) or hypoxic conditions (94% N_2_, 5% CO_2_ and 1% O_2_ at 37 °C). Mouse oral squamous cell carcinoma cell line (SCCVII) was kindly provided by Prof. Shibahara (Tokyo Dental College). SCCVII were cultured in Dulbecco’s modified Eagle’s medium (DMEM; Nissui Pharmaceutical Co., Tokyo, Japan) supplemented with 10% FBS (Biowest, France) and 100 U/mL penicillin-streptomycin (Sigma Aldrich, St. Louis, MI, USA) at 37 °C in 5% CO_2_ atmosphere. All experiments described below were performed with mycoplasma-free cells.

### 2.2. Proliferation Assay

TECs and NECs (1000 cells each) were seeded onto collagen type I-coated 24-well plates (Sumitomo Bakelite Co.). After 24 h, both cell lines were treated with 0, 0.012, 0.12, 1.2 and 6.0 µM of bLF (Morinaga Milk Industry, Tokyo, Japan). Trypsinized cells were counted at 0, 1 and 3 days using Cell Counter (Coulter Z1, Coulter Co., Hialeah, FL, USA).

### 2.3. Wound Healing Assay

Cells (3 × 10^5^) were seeded onto 6 cm collagen type I-coated dishes. After cell seeding for 24 h, a wound area was scratched using a yellow tip. TECs and NECs were treated with 0.012, 0.12 and 1.20 µM of bLF and photographs were taken at 0, 24, 36 and 48 h using an inverted phase-contract microscope (Nikon Instech Co., Ltd., Tokyo, Japan; 4x objective). The wound areas were measured, then the closure areas were calculated and compared to those in the initial wound area.

### 2.4. Migration Assay

After trypsinization, 1.5 × 10^5^ cells of TECs and NECs were resuspended in 100 µL of medium and placed in the upper compartment of a collagen type I-coated 24-well cell culture (Sumitomo Bakelite Co.) insert for 12 and 24 h at 37 °C in a humidified 95% air 5% CO_2_ atmosphere, while 0, 0.012, 0.12 and 1.20 µM of bLF were added in the media. After the incubation, the number of cells that had migrated to the lower side of the membrane were fixed with 10% buffered formalin and stained with hematoxylin. The cells on the upper surface of the filter were wiped off with a cotton swab, and the migration of the cells was determined by counting the migrating cells onto the lower side of the filter through the pores under the microscope at 100× magnification. This assay was performed three times and three fields for counting were randomly selected.

### 2.5. Tube Formation Assay

Basement membrane matrix (BD Matrigel^TM^; BD Biosciences, CA, USA) in 200 µL volume was transferred to each well of a 24-well plate and incubated at 37 °C for 1 h to allow the matrix solution to solidify. Trypsinized TECs and NECs (5 × 10^4^) were seeded, followed by the incubation of 1.2 µM bLF or 10 μM SU6668 (PDGFR/VEGFR2 tyrosine kinase inhibitor: Calbiochem-Merck Millipore, MA, USA) at 37 °C for 9 h. SU6668 is a VEGF, PDGF, or VEGF-R 2 tyrosine kinase inhibitor (TKI) and served as positive control of angiogenesis in the tube formation assay. The number of tube junctions, which is an intersection point of at least 3 tubes, was counted.

### 2.6. Detection of Apoptosis Using Flow Cytometry Analysis

Apoptosis level was measured using the dual-color Annexin V-PE kit (BD Biosciences Pharmigen, Franklin Lakes, NJ, USA) according to the manufacturer’s instructions. TECs and NECs were cultured in EGM-2 MV containing bLF (0.012, 0.12, 1.20 µM) for 3 days. The distribution of early apoptotic cells was analyzed from the dot plot quadrant marker using fluorescence-activated cell sorting (FACS Calibur Flow Cytometer; Becton-Dickinson, San Jose, CA, USA).

### 2.7. Western Blot Analysis

For hypoxic experiments, cells were lysed in 7 M Urea, 10 nmM Tris-HCl (pH 6.8) and 10% glycerol. For TNF-receptor-associated factor 6 (TRAF6) binding to bLF, immunoprecipitates from bLF-pre-treated TECs and NECs were also subjected to Western blotting. Cell lysates were obtained by lysing the cell monolayer with an immunoprecipitation lysis buffer containing 1 M Tris, 5 M NaCl, 0.5 M ethylenediaminetetraacetic acid (EDTA), 100% NoniDet 40 and 100% glycerol. Lysates were incubated on ice for 30 min and centrifuged at 13,200 rpm for 20 min at 4 °C. The supernatants were deemed as a whole lysate. Western blotting was carried out as described previously [30].

### 2.8. Gene Expression Experiments

TECs and NECs cells were seeded in 6 cm dishes (2 × 10^5^) and cultured in EGM-2 MV containing 10% FBS. At 36 h and 72 h after treatment with bLF (0, 0.012, 0.12, 1.20 µM), total RNA was extracted using a RNeasy Mini Kit (Qiagen, Hilden, Germany) according to the manufacturer’s instructions. cDNA was synthesized from 1 µg of total RNA using a ReverTra Dash kit (Toyobo Co., LTD., Osaka, Japan). Aliquots of total cDNA were amplified with GoTaq Green Master Mix (Promega, Madison, WI, USA) and amplifications were performed. The mouse primer pairs used are 5′-GATCATGCCGTCCTTAGAAAA-3′ (forward)/5′-CTGCTTTTTATTTCATGAGGTACATT-3′ (reverse) for *Bcl2*, 5′-CAGGCTGCTGTAACGATGAA-3′ (forward)/5′-AATGCTTTCTCCGCTCTGAA-3′ (reverse) for *Vegfa*, 5′-CGGCAGACCAATACAATCCT-3′ (forward)/5′-TCCGCTGCCTTATAGATGCT-3′ (reverse) for *Flt1*, 5′-GGCGGTGGTGACAGTATCTT-3′ (forward)/5′-GTCACTGACAGAGGCGATGA-3′ (reverse) for *Flk1*, 5′-TGCCATCCCTCAATGTCGAT-3′ (forward)/5′-TGTATGTCCTTCCGCACACT-3′ (reverse) for *Vhl*, 5′-TGGATAGCGATATGGTCAATGT-3′ (forward)/5′-CTCCAAATCTAAATCAGTGTCCTG-3′ (reverse) for *Hif1a*, 5′-GACCAGGTGTTGGACACAGATG-3′ (forward)/5′-AGTCGTTGTCTCCGTCACACTTC-3′ (reverse) for Lipoprotein receptor-related protein-1 (*Lrp1*), 5′-GATCGGGTTGTGTGTGTCTG-3′ (forward)/5′-AGACACCCCAGCAGCTAAGA-3′ (reverse) for *Traf6* and 5′-GCATCCTGGGCTACACTGAG-3′ (forward)/5′-TCCACCACCCTGTTGCTGTA-3′ (reverse) for *Gapdh*. The amplification reaction products were resolved on 1.2% agarose/TAE gels.

### 2.9. Quantitative RT-PCR

Total RNA and cDNA were prepared as above. A one-tenth aliquot of the cDNA was subjected to quantitative RT-PCR (RT-qPCR) using primers (final concentration 200 nM each) and MGB probe (final concentration 100 nM, the Universal Probe Library: UPL, Roche Diagnostics, Tokyo, Japan) sets for *Hif1a*, *Vegfa*, *Flt1*, *Flk1*, and *Gapdh* as an internal control. The mouse primer pair set MGB probes used are 5′-CATGATGGCTCCCTTTTTCA-3′ (forward)/5′-GTCACCTGGTTGCTGCAATA-3′ (reverse)/UPL #18 (Roche) (probe) for *Hif1a*, 5′-CAGGCTGCTGTAACGATGAA-3′ (forward)/5′-GCTTTGGTGAGGTTTGATCC-3′ (reverse)/UPL #9 (Roche) (probe) for *Vegfa*, 5′-ATCGGCCATCATCTGAATGT-3′ (forward)/5′-GCAGTATTCCACGATCACCA-3′/UPL #79 (Roche) (probe) for *Flt1*, 5′-GCTAGCTGTCGCTCTGTGG-3′ (forward)/5′-TTTCTGTGTGCTGAGCTTGG-3′ (reverse)/UPL #58 (Roche) (probe) for *Flk1* and 5′-AAGAGGGATGCTGCCCTTAC-3′/5′-CCATTTTGTCTACGGGACGA-3′/UPL #33 (Roche) (probe) for *Gapdh*. PCR reactions were carried out using a 7500 Real-Time PCR System (Applied Biosystems, Carlsbad, CA, USA) under standard conditions. At least three independent measurements were averaged, and relative gene expression levels were calculated using *Gapdh* expression as the denominator for each sample.

### 2.10. Small Interfering RNA (siRNA) Gene Knockdown

LRP-1 siRNA, TRAF6 siRNA and control siRNA oligos were purchased from Applied Biosystems. siRNA insertion into TECs or NECs was performed as described previously [30] using Lipofectamine RNAiMAX (Invitrogen, Waltham, MA, USA).

### 2.11. In Vivo Experiment

Eight-week-old male C3H/HeN mice (n = 16) were purchased from Charles River Laboratories Japan, Inc. (Yokohama, Japan). Liposomal bLF (LbLF) was obtained from Sunstar Inc. (Osaka, Japan). This study was carried out in strict accordance with the recommendations in the Guide for the Care and Use of Laboratory Animals of the Hiroshima University Animal Research Committee and AVMA Guidelines on Euthanasia. The protocol described below was approved by the Committee on the Ethics of Animal Experiments of the Hiroshima University (Permit Number: A11-141). All mice were housed in a specific pathogen-free-facility in 12 h light-dark cycles with access to water and food ad libitum. At operation, all experimental mice were anaesthetized by intraperitoneal injection of somnopentyl (pentobarbital sodium; 6.48 mg/kg; Kyouritu Seiyaku, Tokyo, Japan) and atropine sulfate (0.05 mg/kg; Mitsubishi Tanabe Pharma Co., Osaka, Japan). Mice were divided into 3 groups: control (C), LbLF 1.2 mM/kg/day and LbLF 6 mM/kg/day. In LbLF groups, LbLF was orally administrated from 7 days before injection of PBS (100 μL) containing SCCVII (10^4^ cells) at the masseter region. Mice were continuously administered LbLF during experimental period. After 3 weeks of transplantation, tumors and surrounding tissues were resected. The samples were fixed with 10% formalin, decalcified in 1 mM PBS (pH 7.4) containing 10% EDTA for 3 weeks at 4 °C, and subsequently embedded in paraffin. Serial sections (4.5 μm) were cut. The sections were stained with hematoxylin-eosin for histological observation.

### 2.12. Immunohistochemical Staining

Immunohistochemical staining was carried out using a Histofine mouse stain kit (mouse tissue, mouse primary antibody; Nichirei Bioscience Inc., Tokyo, Japan). The monoclonal anti-mouse endothelial cell marker CD-31 (PECAM-1) antibody (JC70A, DAKO Japan, Tokyo, Japan, 1:50) was added after antigen retrieval in DAKO antigen retrieval solution (DAKO). Staining was visualized using a DAB Peroxidase (HRP) Substrate Kit (DAKO). Under 200× magnification, CD-31-positive vessels in each sample were counted in 5 different fields. CD-31-positive vessels in adjacent salivary gland tissue, which were considered as normal vessels, were also counted.

### 2.13. Statistical Analysis

Results are reported as mean ± standard deviation (SD). Statistical differences among experimental groups were evaluated by analysis of variance (ANOVA) with the level of significance set at *p* < 0.01 (**) and *p* < 0.05 (*) using SPSS software (IBM, Armonk, NY, USA).

## 3. Results

### 3.1. bLF Significantly Inhibits Angiogenic Ability of TECs but Not NECs

**Figure 1:**
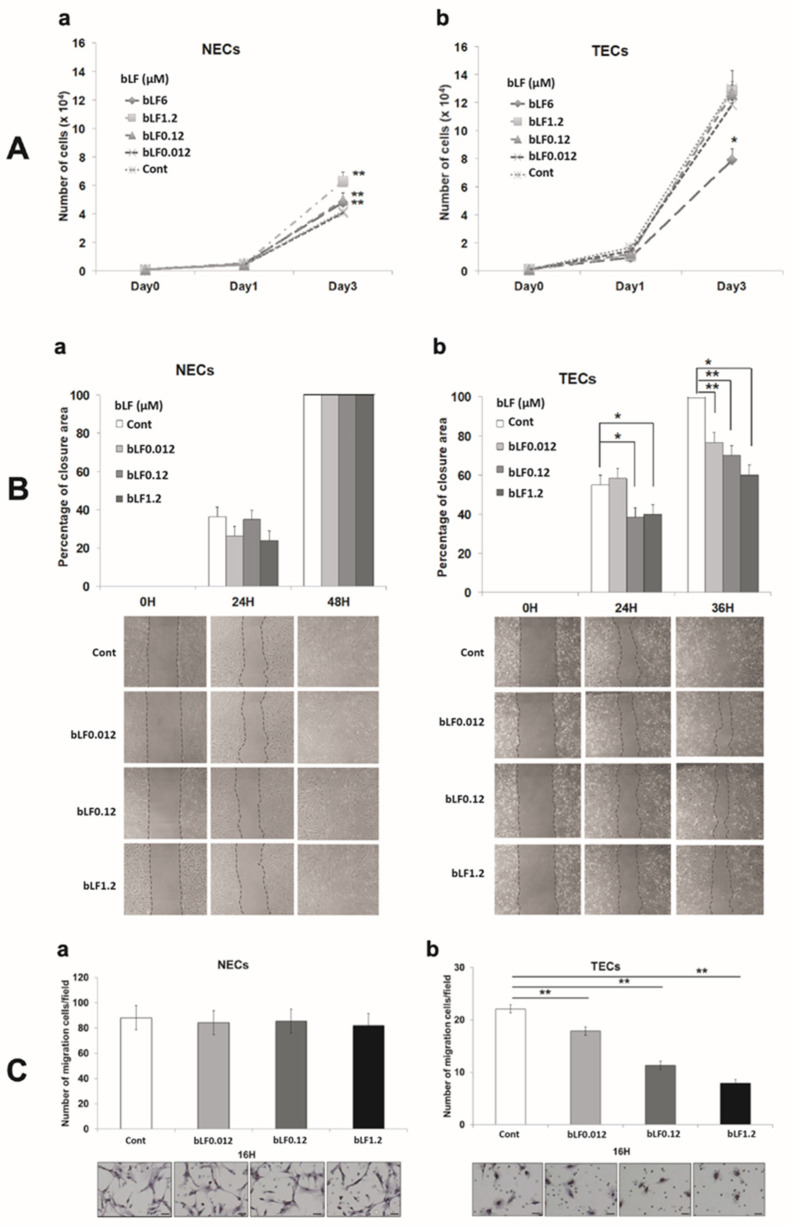
Effect of bovine lactoferrin (bLF) on proliferation and motility of normal endothelial cells (NECs) and tumor endothelial cells (TECs). (**A**) Number of cells treated with bLF (0.012, 0.12, 1.2 μM). (**a**) NECs and (**b**) TECs. (**B**) Data were analyzed by measuring the closure area and compared to the 0H control. Complete closure is represented by 100%. (**a**) NECs and (**b**) TECs. (**C**) Data analyzed by counting the number of cells migrated through membrane pores. (**a**) NECs and (**b**) TECs. Data represent the mean ± standard deviation (SD) of three independent experiments, each performed in triplicate. Statistical significances were determined by one-way analysis of variance (ANOVA). * *p* < 0.05, ** *p* < 0.01.

Endothelial cell activation, proliferation, and migration are required for new capillary formation. The properties of bLF-treated TECs and NECs including proliferation and motility, were examined. bLF at 0.12, 1.2, and 6 μM significantly promoted NECs proliferation (*p* < 0.01) (Figure 1(Aa)). TECs showed a higher proliferation rate than NECs did. Although 6 μM bLF significantly inhibited cell proliferation in TECs (*p* < 0.05) (Figure 1(Ab)), the inhibitory effect of bLF on cell proliferation was limited. The wound-healing assay (Figure 1(Ba) (NECs), Figure 1(Bb) (TECs)) and migration assay (Figure 1(Ca) (NECs), Figure 1(Cb) (TECs)) showed that bLF significantly suppressed the cell motility of TECs in a dose-dependent manner but not in NECs (*p*; * <0.05, ** <0.01).

### 3.2. bLF Suppresses Tube Formation of TECs but Promotes That of NECs

**Figure 2:**
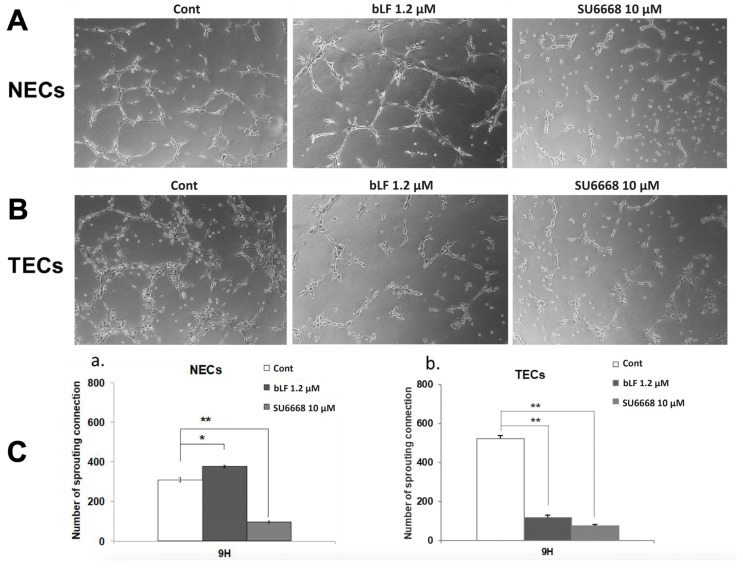
Effect of bLF on tube formation of NECs and TECs. Tube formation assay was conducted in the presence of 1.2 μM bLF or 10 μM SU6668. In vitro assay findings, (**A**) NECs and (**B**) TECs. (**C**) Number of tube junctions in (**a**) NECs and (**b**) TECs. Data represent the mean ± standard deviation (SD) of three-independent experiments, each performed in triplicate. Statistical significances were determined by one-way analysis of variance (ANOVA). * *p* < 0.05, ** *p* < 0.01.

Figure 2A,B shows the representative tissue-structural findings of NECs and TECs. In NECs, a slight increase in tube junctions was observed after being treated with bLF (*p* < 0.05). In contrast, the number of tube junctions in NECs was significantly reduced in the presence of SU6668, an inhibitor of VEGF signaling (*p* < 0.01) (Figure 2A,(Ca)). On the other hand, in TECs, tube junctions were significantly impaired both by bLF and SU6668 treatment (*p* < 0.01) (Figure 2B,(Cb)).

### 3.3. bLF Induces Apoptosis of TECs through Activation of Apoptotic Effector Caspases

**Figure 3:**
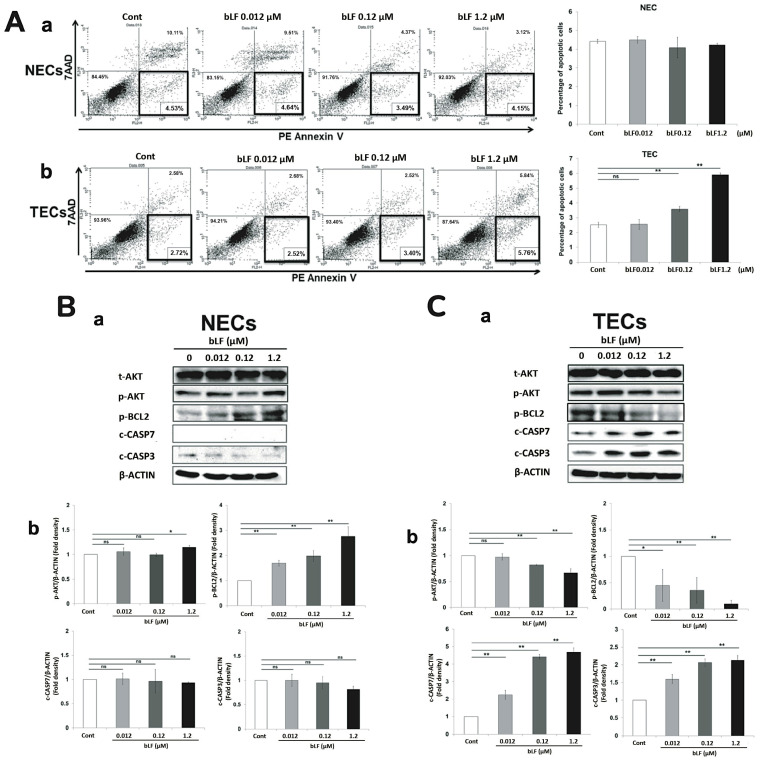
Effect of bLF on apoptosis of NECs and TECs. (**A**) The percentage of early apoptotic cells in NECs (**a**) and TECs (**b**). Effect of bLF on apoptosis-related proteins in TECs (**C**) and NECs (**B**). Both cells were cultured with bLF treatment (0.012, 0.12, 1.2 μM) for 3 days. Protein expression was analyzed by Western blotting (**a**). Semi-quantitative analysis was also conducted (**b**). Data quantification was performed using imageJ software (National Institutes of Health, US). Data represent the mean ± standard deviation (SD) of three-independent experiments, each performed in triplicate. Statistical significances were determined by one-way analysis of variance (ANOVA). ns: not significant; * *p* < 0.05, ** *p* < 0.01.

Dual-color flow cytometry assay using PE Annexin V identifying early apoptotic cells with exposed phosphatidylserine and 7AAD identifying late apoptotic cells with cell membrane destruction showed that bLF did not induce apoptosis of NECs (Figure 3(Aa)). In contrast, bLF caused a slight but significant increase in early apoptotic cells in TECs (Figure 3(Ab)). To clarify the underlining mechanisms of bLF-induced TEC-specific apoptosis, proteins related to apoptosis were analyzed by Western blotting. The results showed no significant changes were detected in caspase-3 and -7 expression, but, interestingly, increases in AKT and BCL2 phosphorylation levels were observed in NECs treated with bLF (Figure 3(Ba,Bb)). In contrast, the inhibition of AKT, BCL2 phosphorylation and induction of cleaved caspase-3 and -7 in TECs are evident upon bLF treatment (Figure 3(Ca,Cb)).

### 3.4. bLF Is Internalized in NECs and TECs through LRP-1

**Figure 4:**
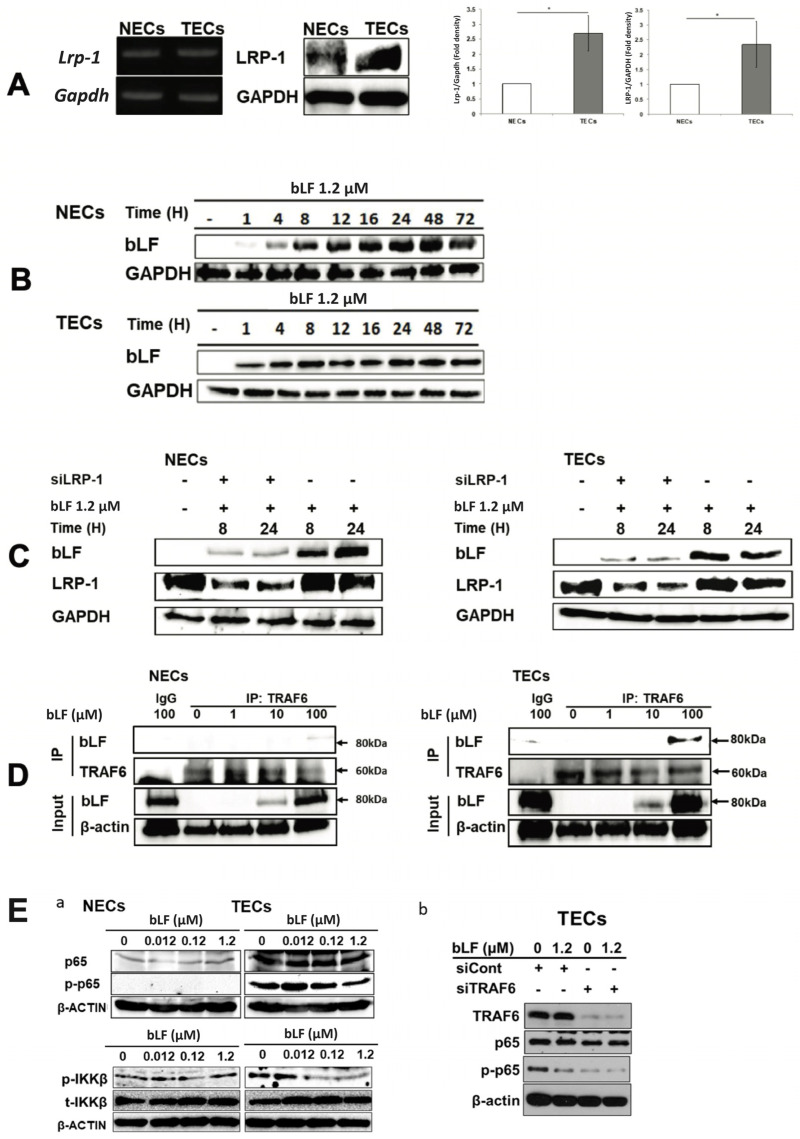
bLF receptor and its role as an internalization factor for bLF in NECs and TECs. (**A**) LRP-1 mRNA and protein expression in NECs and TECs. Semi-quantitative analysis was also conducted. Data quantification was performed using imageJ software (National Institutes of Health, US). Data represent the mean ± standard deviation (SD) of three-independent experiments, each performed in triplicate. Statistical significances were determined by one-way analysis of variance (ANOVA). * *p* < 0.05. (**B**) Detection of internalized bLF by Western blotting in NECs and TECs at 1, 4, 8, 12, 16, 24, 48 and 72 h after 1.2 μM bLF treatment. (**C**) LRP-1 expression and bLF internalization by Western blotting in siLRP-1 NECs and TECs at 8 and 24 h after 1.2 μM bLF treatment. (**D**) Immunoprecipitation of bLF and TRAF6. (**E**) p-p65 protein levels by Western blotting in NECs and TECs after 0.012, 0.12 and 1.2 μM bLF treatment (**a**). p-p65 expression level in siTRAF6 transfected TECs with 1.2 μM bLF treatment (**b**).

A previous report showed that bLF was mainly endocytosed through LRP-1 [30]. Here, expression levels of LRP-1 mRNA and protein in TECs were significantly higher than those in NECs (Figure 4A) and internalized bLF with time (Figure 4B). The amount of intracellularly distributed bLF in both siLRP-1 NECs and TECs was drastically lesser than those in the control (Figure 4C), indicating LRP-1 is the main receptor for bLF endocytosis both in TECs and NECs. Previously, it was reported that bLF endocytosed by LRP-1 would bind to TRAF6, a key molecule of the NF-κB pathway [30]. To confirm bLF–TRAF6 binding, immunoprecipitation was performed. Western blotting of immunoprecipitates from bLF-treated TECs and NECs revealed that bLF directly bound to endogenous TRAF6 (Figure 4D). It is also reported that bLF binds to TRAF6 and markedly inhibits p-p65 expression of the NF-kB pathway in osteoblastic cells with LPS-stimulation [30]. Here, constitutive expression of NF-kB pathway molecules in NECs and TECs were examined. Interestingly, TECs constitutively expressed a higher amount of total p65, p-p65 and p-IKKβ. On the other hand, only a slight amount of p65 was detected in NECs. bLF dose-dependently reduced p-p65 and p-IKKβ levels in TECs, but not in NECs (Figure 4(Ea)). Moreover, we confirmed an effect of TRAF6 silencing on TECs with bLF stimulation. In siCont TECs with strong expression of TRAF6, bLF induced a marked reduction in the p-p65 expression level, while with siTRAF6 TECs, there was no difference in p-p65 expression with or without bLF treatment (Figure 4(Eb)). The findings indicate that bLF–TRAF6 binding is essential for the inhibition of the NF-kB pathway caused by bLF.

### 3.5. bLF down Regulates Proangiogenic Genes and Its Transcription Factor through Inhibiting NF-κB Activation in TECs

NF-κB signaling is also known to up-regulate HIF-1α expression through the binding of several NF-κB subunits to *Hif1a* promoter resulting in the induction of *Vegfa* [31]. Here, we investigated the effect of bLF on HIF-1α regulatory genes.

**Figure 5:**
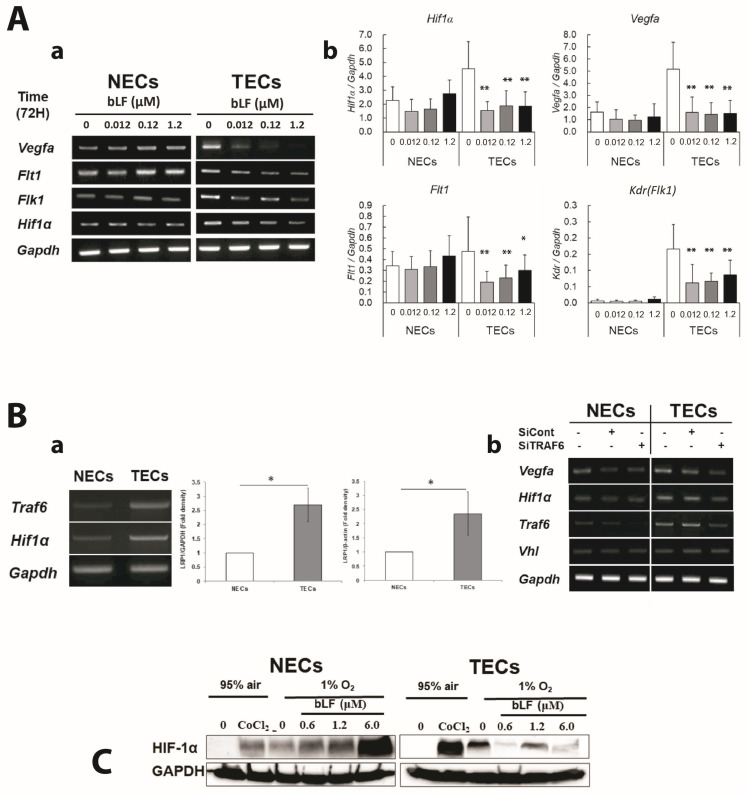
Effect of bLF on the NF-κB pathway, HIF-1α, and its regulated molecules. (**A**) mRNA levels of pro-angiogenic genes (i.e., *Vegfa*, *Flt1* and *Flk1*) and *Hif1a* detected by RT-PCR (**a**) and RT-qPCR (**b**). Data represent the mean ± standard deviation (SD). Statistical significances were determined by one-way analysis of variance (ANOVA). * *p* < 0.05, ** *p* < 0.01. (**B**) Expression of *Traf6* and *Hif1a* in NECs and TECs examined by RT-PCR. Semi-quantitative analysis was also conducted. Data quantification was performed using ImageJ software (National Institutes of Health, US). Data represent the mean ± standard deviation (SD) of three-independent experiments, each performed in triplicate. Statistical significances were determined by one-way analysis of variance (ANOVA). * *p* < 0.05 (**a**). *Vegfa*, *Hif1a*, *Traf6*, and *Vhl* mRNA levels examined in siTRAF6 transfected NECs and TECs (**b**). (**C**) HIF-1α levels detected by Western blotting after bLF treatment under hypoxia condition induced by CoCl_2_ or 1% O_2_ cultivation.

Figure 5A show the effect of bLF on *Hif1a* and its regulated genes mRNA in NECs and TECs by RT-PCR (Figure 5(Aa)) and RT-qPCR (Figure 5(Ab)), respectively. Higher expression of *Vegfa*, *Flt1*, *Flk1* and *Hif1a* were observed in TECs compared with those in NECs, and bLF markedly downregulated those expressions in TECs. In contrast, bLF did not show any effects on NECs except for the slight up-regulation of *Flt1*.

Recently, TRAF6 has been reported to contribute to tumor angiogenesis by up-regulating HIF-1α expression [32]. Interestingly, *Traf6* and *Hif1a* mRNA were expressed at higher levels in TECs than in NECs (Figure 5(Ba)), suggesting the possible role of these molecules in the specific inhibitory effect of bLF on the responses of TECs. To further clarify the relationship between TRAF6 and HIF-1α, the effect of specific siRNA for *Traf6* (siTRAF6) was explored. In siTRAF6 cells, the mRNA levels of *Hif1a* and *Vegfa* were downregulated. There were no differences in *Vhl* mRNA expression level between NECs and TECs. These results indicate the importance of TRAF6 in the regulation of HIF-1α at the transcriptional level (Figure 5(Bb)).

Under normoxic conditions, HIF-1α protein could not be detected in either NECs or TECs because it was quickly degraded. Under hypoxia-mimic conditions with COCl_2_ (chemical inducer of HIF-1α) treatment or hypoxia (1% O_2_), TECs expressed a higher amount of HIF-1α protein than NECs did. Next, the effects of bLF on HIF-1α protein expression in both cell types under hypoxic condition were analyzed. bLF decreased HIF-1α protein in TECs, whereas it increased in NECs (Figure 5C).

### 3.6. Effects of Orally Administrated LbLF on Microvascular Density in the In Vivo OSCC Model

CD-31 is expressed on all vascular endothelial cells and is involved in cell adhesion and signal transduction. It is reported that tumor-associated endothelial cells express typical endothelial cell markers, including CD31 [28]. Therefore, we evaluated the number of CD-31-positive TEC-vessels in tumor xenograft tissues and NEC-vessels in adjacent salivary gland tissue, between LbLF treated and non-treated animals.

**Figure 6:**
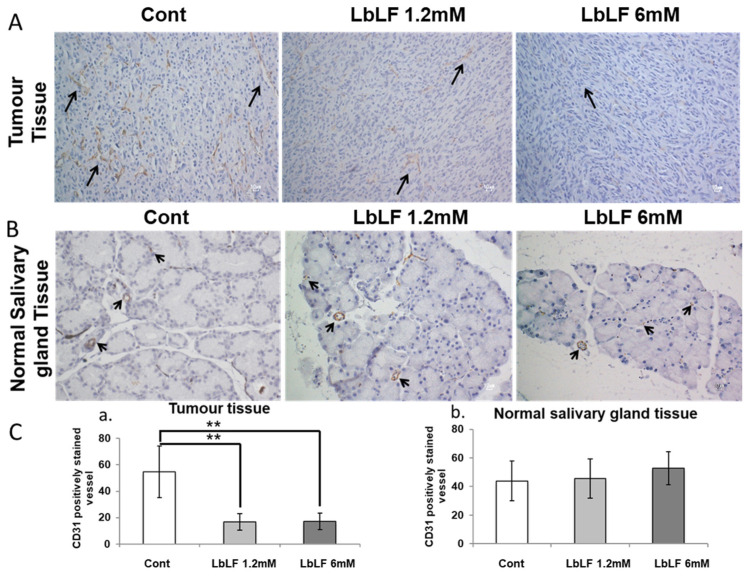
CD-31 immunohistochemical staining in tumor and surrounding salivary gland tissues. (**A**) Transplanted oral squamous cell carcinoma cell (OSCC) tissue. (**B**) Salivary gland. Black arrows show CD-31-positive capillaries. (**C**) Number of CD-31-positive capillaries in tumor tissue (**a**) and normal salivary gland tissue (**b**). Data represent the mean ± standard deviation (SD). Statistical significances were determined by one-way analysis of variance (ANOVA). ** *p* < 0.01.

Within tumor xenograft tissues, numerous CD-31-positive vessels were observed. On the other hand, the number of CD-31-positive vessels in LbLF treatment groups was scant and the staining intensity was weaker (*p* < 0.01) (Figure 6A,(Ca)). However, thin-walled vessels stained with CD-31 in normal salivary glands of both the control and LbLF-treated groups were evenly interspersed (Figure 6B,(Cb)).

## 4. Discussion

To progress, tumors require a source of nutrition and oxygen to form masses greater than 1–2 mm. Tumor angiogenesis is inevitable for the acquisition of sufficient oxygen supply [33,34]. It is well known that high VEGFA and HIF-1α expression levels are associated with a worse outcome in a wide array of malignancies through promoting tumor angiogenesis [35]. It has been reported that bLF suppresses lung or colon cancer growth through decreasing the expression of VEGFA and pro-inflammatory cytokines [21,35,36]. Therefore, bLF may inhibit tumor angiogenesis through VEGFA inhibition. However, the inhibitory mechanisms of bLF on tumor angiogenesis and tumor endothelial cells have not been fully elucidated. To establish a new bLF therapy targeting tumor angiogenesis, we aimed to clarify the differences in bLF effects between normal and tumor endothelial cells. In the present study, unique endothelial cells (TECs and NECs), representative for tumor endothelial cells and normal endothelial cells, were used. A previous study reported that TECs are cytogenetically abnormal, express higher VEGFA, FLK1, and HIF-1α, and show higher migration and proliferation than NECs [28,37]. Here, we confirmed that the higher expression of HIF-1α, VEGFA, and FLK1 in TECs leads to higher cell proliferation and migration potential.

In the present study, bLF specifically inhibited the angiogenic abilities of TECs such as proliferation, migration, and tube formation. Interestingly, we confirmed that TECs have a high expression of TRAF6 and NF-κB p65. Recent studies indicated that TRAF6 plays an important role in cancer [32]. Our previous study showed that bLF, which is mainly endocytosed through LRP-1, inhibited the lipopolysaccharide (LPS)-induced NF-κB pathway in an osteoblastic cell line through binding to TRAF6 [30]. Here, we observed that TECs expresses LRP-1. Moreover, the amount of intracellularly distributed bLF in siLRP-1 transfected TECs was remarkably less than that in control TECs, suggesting the role of LRP-1 as an internalization receptor for bLF. The immunoprecipitation of bLF-treated TECs revealed that bLF directly bound to endogenous TRAF6, leading to the inhibition of the NF-kB pathway. Moreover, knockdown of TRAF6 in TEC cells, which express TRAF6 abundantly, prevented the LF-induced reduction in p-p65 expression, indicating that TRAF6–bLF binding is critical for bLF-induced anti-angiogenetic action.

The transcription factors NF-κB and HIF-1α are frequently activated in tumors, involved in tumor growth and progression, and resistant to chemotherapy through their ability to up-regulate the expression of tumor-promoting cytokines and survival genes. It has been reported that NF-κB upregulates HIF-1α expression by binding to the *Hif1a* gene promoter. NF-κB depletion results in the reduction in the basal levels of *Hif1a* mRNA [32]. The present study showed that down-regulation of NF-κB by bLF–TRAF6 binding induced the reduction of HIF-1α protein in TECs. To further highlight the relationship between TRAF6 and HIF-1α, TRAF6 silencing with siRNA was carried out. The mRNA level of *Hif1a* and its downstream target *Vegfa* were downregulated in siTRAF6 transfected TECs. These results indicated the important role of TRAF6 in the regulation of HIF-1α expression. It was reported that TRAF6 binds to HIF-1α and increases HIF-1α polyubiquitination, promoting in vivo tumor angiogenesis [32]. These findings suggest that bLF–TRAF6 binding in TECs may inhibit the NF-κB pathway and, therefore, HIF-1α and VEGF-A expression.

Here, we emphasized that TRAF6 and NF-κB p65 were overexpressed in TECs but not in NECs. The overexpression of TRAF6 and p65 could explain why bLF specifically inhibits HIF-1α expression and the angiogenic abilities of TECs. In this study, NF-κB appeared to be one of the main pathways in the regulation of HIF-1α and VEGFA expression in TECs, but not in NECs. In fact, bLF inhibited proliferation, motility, and tube formation only in TECs. Conversely, bLF treatment induced HIF-1α expression and proliferation in NECs. It was reported that the AKT pathway is involved in the synthesis and accumulation of HIF-1α, and that HIF-1α–induced VEGF activated the AKT pathway [38]. We confirmed that AKT phosphorylation was up-regulated only in NECs treated with bLF. Although our results may explain why bLF specifically acts as an anti-angiogenic drug in TECs, further experiments are needed to fully elucidate the underlying mechanisms.

It is known that BAX/BAK initiate apoptosis by allowing cytochrome-c to escape into the cytoplasm and activate the pro-apoptotic caspase cascade. The anti-apoptotic BCL2 inhibits cytochrome-c to attenuate activation of the cytoplasmic caspase cascade [39]. In the present study, bLF slightly but significantly up-regulated apoptosis of TECs by inhibiting AKT and BCL2 phosphorylation and inducing the caspase cascade. On the other hand, in NECs treated with bLF, AKT phosphorylation was evident, and no changes were detected in the levels of cleaved caspase-3 and -7. Additionally, BCL2 phosphorylation was upregulated.

Recently, targeted therapies using monoclonal antibodies that antagonize the formation of new blood vessels have been developed. One example is bevacizumab, a genetically engineered humanized monoclonal immunoglobulin-G antibody that traps VEGFA and blocks the VEGF receptor signaling in endothelial cells. However, bevacizumab was also associated with major risks of thrombosis, fatal hemorrhage and visceral perforation [36] due to its suppression of both tumor and physiological angiogenesis. In the OSCC mice model, we administered LbLF and observed considerable CD-31-positive micro vessel density (MVD), which is evident in small vessels with immature endothelium [40], in tumor areas in comparison with that in normal areas. The result showed MVD reduction in the tumor area, but no significant difference was observed in the normal area compared to the control, suggesting that LbLF could be a specific therapy targeting tumor angiogenesis without any harmful effects on physiological angiogenesis. These results strongly support the safety of bLF used as an anti-angiogenic agent in cancer therapy.

## 5. Conclusions

In this study, we demonstrated that bLF treatment inhibited angiogenic phenotypes and induced apoptosis of TECs both in in vitro and in vivo studies. Remarkably, angiogenesis inhibition was evident only in TECs. We further found that TRAF6 and p65 were expressed at higher levels in TECs than in NECs, and that bLF bound to TRAF6. bLF specifically suppressed tumor angiogenesis through the inhibition of the NF-κB pathway by binding to TRAF6, suppressing p65 phosphorylation, the reduction of HIF-1α and its target gene expressions. Collectively, our results suggest that bLF could be used as a beneficial anti-angiogenic agent for OSCC and other tumors through its TEC-specific anti-angiogenic action.

## Data Availability

Not applicable.
